# EGFR confers radioresistance in human oropharyngeal carcinoma by activating endoplasmic reticulum stress signaling PERK‐eIF2α‐GRP94 and IRE1α‐XBP1‐GRP78

**DOI:** 10.1002/cam4.1862

**Published:** 2018-11-09

**Authors:** Miao Zhang, Ning Han, Yuanjun Jiang, Jie Wang, Gaiyan Li, Xintong Lv, Guang Li, Qiao Qiao

**Affiliations:** ^1^ Department of Radiation Oncology The First Hospital of China Medical University Shenyang Liaoning China; ^2^ Department of Urology The First Hospital of China Medical University Shenyang Liaoning China

**Keywords:** EGFR, ERS, oropharyngeal carcinoma, radiation, radioresistance

## Abstract

The activation of epidermal growth factor receptor (EGFR) is associated with radioresistance in malignant tumors. Specifically, radiation can destroy endoplasmic reticulum (ER) homeostasis to induce ER stress (ERS). However, the effect of EGFR‐mediated regulation of ERS signaling pathway on radiosensitivity has not yet been reported. The present study showed that silencing EGFR increased radiosensitivity of both radiosensitive and radioresistant oropharyngeal squamous cell carcinoma (OSCC) cells by inhibiting ER stress signaling (PERK‐eIF2α‐GRP94 and IRE1α‐XBP1‐GRP78). This effect was abolished by pretreatment with EGF, however. In addition, knockdown of EGFR in OSCC cells inhibited DNA double‐stand break repair and autophagy while increased radiation‐induced apoptosis. Conversely, activating ERS inhibited the aforementioned functions. Furthermore, EGF increased ER stress‐independent ERK and AKT signaling upon irradiation of OSCC cells. Immunohistochemical analysis of 80 tissue samples from OSCC patients showed that co‐expression of EGFR and PERK was associated with poor prognosis. It thus appears EGFR confers radioresistance in OSCC by activating ER stress signaling. These results suggested that the cooperative effects of radiotherapy and EGFR‐targeted inhibitor therapy can be further improved by inhibiting PERK‐eIF2α‐GRP94 and IRE1α‐GRP78 in non‐response oropharyngeal carcinoma patients.

## 1 INTRODUCTION

The incidence of oropharyngeal carcinoma has been increasing worldwide with nearly 136 000 new cases diagnosed annually.^1^ Currently, combined radiotherapy and chemotherapy regimen is preferred for patients with advanced oropharyngeal squamous cell carcinoma (OSCC) because it improves local cancer control and overall survival (OS) than radiotherapy alone. However, the acute and long‐term adverse effects of the combined approach limit their clinical application. In recent years, therapeutic drugs targeting the epidermal growth factor receptor (EGFR) such as cetuximab and nimotuzumab have been used to treat malignant tumors of the head and neck because of their low adverse effects. Combined EGFR‐targeted therapy and radiotherapy increases the median survival of patients with head and neck malignant tumors from 29.3 months with radiotherapy alone to 49 months without increasing adverse effects such as oral mucositis and dysphagia.^2^ However, 50% of patients will develop local recurrence.^3^ Therefore, it is reasonable to deduce that a portion of head and neck squamous cell carcinoma tumors may present radioresistant, even with suppressed EGFR.

EGFR activates several signaling pathways including extracellular signal‐regulated kinase (ERK) and protein kinase B (AKT), thereby promoting tumor cell proliferation, invasiveness, and survival.^4^ In addition, EGFR modulates tumor cell sensitivity to chemotherapy and radiotherapy.^5^, ^6^ However, the mechanisms determining radioresistance have not been fully established. Several stimuli like abnormal glucose metabolism, radiation, drugs, and inflammation alter endoplasmic reticulum (ER) homeostasis resulting in aggregation of misfolded proteins in the ER This induces ERS and activates the unfolded protein response (UPR), which protects tumor cells from radiation and drug‐induced stress.^7^ Therefore, inhibition of the ERS increases the radiosensitivity of nasopharyngeal carcinoma^8^ and breast cancer^9^ through radiation‐induced DNA double‐strand break repair,^9^ autophagy,^9^ and apoptosis.^8^ In accordance with others, we demonstrated that ER stress confers radioresistance in OSCC.^10^, ^11^ However, the role of EGFR‐regulated ERS signaling on the radiosensitivity of malignant tumors has not yet been reported. Therefore, in this study, we investigated the role of EGFR‐related ERS signaling on radioresistance in OSCC.

## 2 MATERIALS AND METHODS

### 2.1 Cell culture, reagents, and the establishment of radioresistant OSCC cells

The human HPV‐negative OSCC cell lines FaDu and Detroit562 were cultured in minimum essential medium containing 10% heat‐inactivated fetal bovine serum, 100 U/mL penicillin, and 100 µg/mL at 37°C and 5% CO_2_.

The radioresistant FaDu and Detroit562 cell lines were established according to a previously published protocol.^12^ Briefly, 1000 cells/cm^2^ FaDu and Detroit562 cells were seeded in six‐well plates and allowed to attach for 24 hours before receiving 4 and 3 Gy of irradiation, respectively. The cells were then cultured for 10‐14 days to form colonies. The culturing and irradiation process was repeated four times with single‐cell clones. The resultant radioresistant cells are denoted as FaDuR and Detroit562R, whereas non‐irradiated parental cells are denoted as FaDuP and Detroit562P.

Ly294002, 3‐Methyladenine (3‐MA), and salubrinal were all purchased from Sigma (St Louis, MO, USA). Tunicamycin was purchased from Abcam (Cambridge, UK). Erbitux (cetuximab) was purchased from Merck (Darmstadt, Germany).

### 2.2 Cell transfections

The cells were transfected with ON‐TARGETplus SMARTpool EGFR, IRE1 (ERN1), and PERK (EIF2AK3) siRNAs as well as ON‐TARGETplus Non‐targeting siRNA #1 (Dharmacon, Thermo Fisher Scientific, Waltham, MA, USA) with DharmaFECT 1 transfection reagent.

### 2.3 Western blotting

Total cellular protein lysates were prepared with Pierce lysis buffer (Pierce, Rockford, IL, USA) and quantified by the Bradford method. Equal amounts of total protein (20 µg) were loaded onto 10% SDS‐PAGE gels and electrophoresed at 100 V for 1 hour. Then, the separated proteins were transferred onto PVDF membranes (Millipore, Billerica, MA, USA) at 80 V for 2 hours. The membranes were blocked in 5% non‐fat milk in 1× TBST and then incubated with primary antibodies against EGFR (1:500; Santa Cruz, USA), PERK, IRE1α, ATF 6, (1:1000; Abcam), phospho‐eIF2α, GRP78, GRP94, PDI, ERO1‐Lα, CHOP, phospho‐ATM, DNA‐PK, LC3B, Atg3, cleaved caspase 3, cleaved PARP, and β‐actin (1:1000; Cell Signaling Technology, Boston, MA, USA) at 4°C overnight. After washing with 1× TBST, the membranes were incubated with secondary anti‐mouse or anti‐rabbit IgG HRP‐linked antibodies (1:5000; Cell Signaling Technology) at room temperature for 2 hours. The blots were developed with Target LumiGLO (Cell Signaling Technology) and photographed with DNR BioImaging System (DNR, Israel).

### 2.4 Quantitative real‐time polymerase chain reaction (qRT‐PCR)

Total RNA was extracted with the RNeasy Mini reagent kit (Qiagen, Valencia, CA, USA) according to manufacturer's instructions. Reverse transcription was performed with the M‐MLV reverse transcription reagent kit (Invitrogen, Carlsbad, CA, USA). The PERK and IRE1 mRNA levels were quantitated by TaqMan analysis (Applied Biosystems, Carlsbad, CA, USA).^13^

The following primers were used for qPCR: PERK forward primer, 5′‐CATCCAGCCTTAGCAAACCAGA‐3′; PERK reverse primer, 5′‐AGGAACTGTTTCCATGCTTTCAC‐3′; IRE1 forward primer, 5′‐TTGTCATCGGCCTTTGCAGATA‐3′; IRE1 reverse primer, 5′‐CAGTGAGGCCGCATAGTCAAAGTA‐3′; β‐actin, forward primer, 5′‐TGGCACCCAGCACAATGAA‐3′; and β‐actin reverse primer, 5′‐CTAAGTCATAGTCCGCCTAGAAGCA‐3′.

### 2.5 Colony assay

We seeded 1 × 10^2^‐1 × 10^4^ cells in six‐well plates overnight and then irradiated them in a SIEMENS linear accelerator (SIEMENS Medical Systems, Germany) with doses of 0, 2, 4, and 6 Gy at 2 Gy/min. The cells were then continuously cultured for 10‐14 days with fresh medium added every 24 hours. The cells were fixed in methanol and stained with 5% crystal violet, and colonies with >50 cells were counted. The dose survival curve was plotted using the classic multi‐target single hit model, survival fraction (SF) = 1 − (1 − e^−D/D0^) *N*. Each point on the survival curve represented the mean surviving fraction from at least three independent experiments. The mean lethal dose (D0) is the dose required to reduce the fraction of surviving cells to 37% of its previous value, quasi‐threshold dose (Dq) is the repair capacity of the cells after radiation, and *N* is the extrapolation number. From the survival curve, D0, Dq, survival fraction at 2 Gy (SF2), and sensitivity enhancement ratio (SER) (SER = D0 control group/D0 combination group) were calculated.

### 2.6 Flow cytometry

Cells were seeded in six‐well plates for 12 hours and then treated with 20 µmol/L Ly294002 and 5 mmol/L 3‐MA for 12 hours followed by 5 Gy of irradiation. Then, the cells were harvested after 48 hours and stained with Annexin V using Annexin‐Green Apoptosis cell detection reagent kit (Cell Signaling Technology) according to manufacturer's instructions. The cells were then subjected to flow cytometry in FACS Calibur BD (BD Biosciences, San Jose, CA, USA), and the percentage of Annexin V^+^ (apoptotic) cells were determined for each group of cells.^13^

### 2.7 Immunofluorescence

For detection of residual DNA double‐strand breaks and autophagy, the γ‐H2AX and LC3B foci assay has been described in detail in our previous study.^11^

### 2.8 CCK‐8 cell proliferation assay

Cell proliferation was analyzed with the Cell Counting Kit‐8 (CCK‐8) kit (Dojindo, Gaithersburg, MD, USA) according to the manufacturer's instructions as described in our previous study.^14^

### 2.9 Immunohistochemistry

Tumor sections from 80 HPV‐negative OSCC patients that received radical radiotherapy with or without chemotherapy at our hospital between 2005 and 2011 were obtained. All recruited patients provided informed consent for the study.

The sections were stained with the Elivision staining kit (Maixin Co., Fuzhou, China) according to manufacturer's instruction. Briefly, the sections were incubated with primary PERK and IRE1α (1:100 dilution; Abcam) as well as EGFR (1:50 dilution; Santa Cruz, USA) antibodies at 4°C overnight, and then further processed with the 3,3′‐diaminobenzidine (DAB) kit (Maixin Co.) as described in our previous study.^14^ Two independent blinded investigators randomly examined all tumor slides. PERK and IRE1α staining was cytoplasmic, whereas EGFR staining was both cytoplasmic and nuclear. A semiquantitative scoring was used as previously described.^15^ The scoring system was as follows: 0, no staining; 1, weak staining; 2, moderate staining; and 3, strong staining. The scoring of the specimen based in the percentage of stained tumor cells was as follows: 0, <10%; 1, 10%‐30%; 2, 30%‐60%; and 3, >60%. The sum of both scores was the final score for each tumor sample, which was between 0 and 6. Samples with a final score ≤2 were considered negative staining, whereas those with a final score of 3‐6 were considered positive.

### 2.10 Statistical analysis

Data were expressed as the mean ± SD. Kaplan‐Meier analysis was used to determine OS. The expression of PERK, IRE1α, and EGFR in oropharyngeal carcinoma tissues was analyzed using Spearman correlation, and differences between groups were compared using the *t* test. Two‐sided *P* values <0.05 indicated a significant difference. SPSS13.0 software was used for statistical analyses.

## 3 RESULTS

### 3.1 Differential EGFR activation after irradiation in radioresistant OSCC cell lines

**Figure 1:**
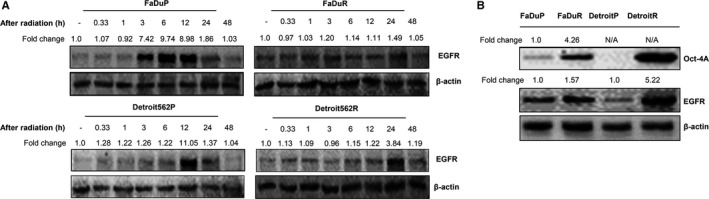
EGFR levels in irradiated OSCC cells. A, EGFR expression in OSCC (FaDuP, FaDuR, Detroit562P, and Detroit562R) cells at different time points (20 min, 1, 3, 6, 12, 24, and 48 h) after 5 Gy of radiation. B, Oct‐4a and EGFR expression in FaDuP, FaDuR, Detroit562P, and Detroit562R cells. As shown, their expression was higher in radioresistant FaDuR and Detroit562R cells than in FaDuP and Detroit562P cells. The bands were quantified with ImageJ software and normalized to a loading control, β‐actin. N/A = not applicable

Similar to previous study,^16^ we observed a time‐dependent increase in EGFR levels upon X‐ray irradiation of OSCC (Detroit562 and FaDu) cells (Figure 1A). In the parental (Detroit562P and FaDuP) cells, EGFR levels increased at 20 minutes after irradiation, peaked at 6‐12 hours, and decreased after 48 hours. But, EGFR levels in the radioresistant Detroit562R and FaDuR cells increased at 3‐6 hours after irradiation, peaked at 24 hours, and persisted until 48 hours.

We observed increased expression of OCT‐4A, a tumor stem cell marker in the radioresistant FaDuR and Detroit562R cells only (Figure 1B). Radioresistant OSCC tumors exhibit tumor stem cell‐like characteristics,^12^ and EGF induces stem cell‐like characteristics in oral cancer cells.^17^ We observed higher EGFR expression in FaDuR and Detroit562R cells than in the parental FaDuP and Detroit562P cells (Figure 1B). These results suggested that irradiation induced EGFR expression in OSCC cells and its persistent overexpression was associated with radioresistance.

### 3.2 EGFR increases radioresistance in oropharyngeal carcinoma cells

**Figure 2:**
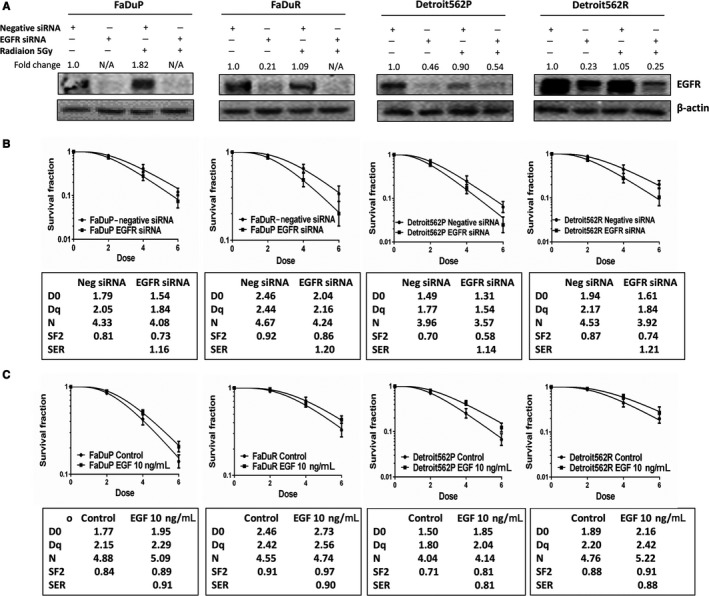
Role of EGFR in the radiosensitivity of human OSCC cells. A, EGFR protein levels in control and EGFR siRNA transfected OSCC (FaDuP, FaDuR, Detroit562P, and Detroit562R) cells 12 h after 5 Gy of irradiation. B, Colony formation assay showing the number of colonies in control and EGFR siRNA transfected OSCC (FaDuP, FaDuR, Detroit562P, and Detroit562R) cells after 0, 2, 4, and 6 Gy irradiation. The cells were then continuously cultured for 10‐14 d and then analyzed. The radiation parameters fitted to classic multi‐target single hit model as shown in the frames under the corresponding figures. C, Colony formation assay showing the number of colonies in control and cells pre‐treated with 10 ng/mL of EGF followed by irradiation after 2 h. As shown, EGF treatment increased the number of OSCC cell colonies after irradiation and fitted to classic multi‐target single hit model as shown in the frames

Next, we assessed the association between EGFR overexpression and radioresistance in oropharyngeal carcinoma cells. We observed that EGFR siRNA transfected OSCC cell lines downregulated EGFR protein (Figure 2A). Moreover, EGFR siRNA transfected OSCC cells showed diminished colony formation after irradiation than control siRNA transfected OSCC cells (Figure 2B). This confirmed that EGFR silencing increased the radiosensitivity of OSCC cells. This effect was more pronounced in radioresistant OSCC cells with the SER for FaDuP, FaDuR, Detroit562P, and Detroit562R cells being 1.16, 1.20, 1.14, and 1.21, respectively. Pre‐treatment of OSCC cells with EGF increased colony formation after irradiation and induced radioresistance (Figure 2C). This confirmed that EGF‐EGFR activation was necessary for radioresistance in OSCC.

### 3.3 EGF‐EGFR activates ERS signaling in OSCC cell lines upon irradiation

**Figure 3:**
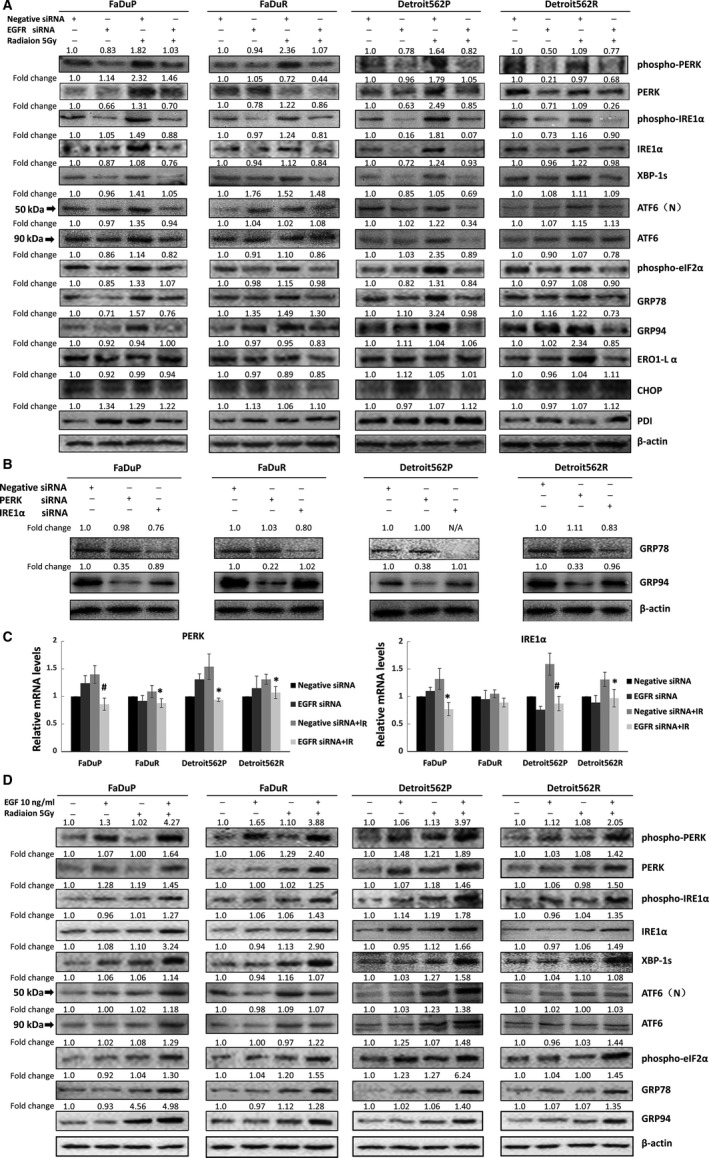
EGF‐EGFR activates ERS signaling. A, Total and phosphorylated PERK, eIF2α and IRE1α as well as GRP78, GRP94, and spliced XBP‐1 in control and EGFR siRNA transfected FaDuR, Detroit562R, FaDuP, and Detroit562P cells, 12 h after 5 Gy of irradiation. Also shown is total ATF6 (90 kDa) and cleaved ATF6 (50 kDa) in FaDuR, Detroit562R, FaDuP, and Detroit562P cells, 12 h after 5 Gy of irradiation. B, GRP94 and GRP78 protein levels in control and PERK and IRE1α siRNA transfected FaDuR, Detroit562R, FaDuP, and Detroit562P cells. C, Quantitative real‐time PCR analysis of PERK and IRE1α mRNA in control and EGFR siRNA transfected FaDuR, Detroit562R, FaDuP, and Detroit562P cells 1 h after 5 Gy of irradiation. As shown, EGFR silencing inhibited irradiation‐induced PERK and IRE1α mRNA expression. Note: *P < 0.05 and ^#^ *P* < 0.01 compared to the irradiated group. D, Total and phosphorylated PERK, eIF2α and IRE1α as well as GRP78, GRP94, and spliced XBP‐1 in control and EGFR siRNA transfected FaDuR, Detroit562R, FaDuP, and Detroit562P cells pre‐treated with 10 ng/mL EGF followed by 5 Gy of irradiation. Also shown is total and cleaved ATF6 in FaDuR, Detroit562R, FaDuP, and Detroit562P cells treated with 10 ng/mL EGF followed by 5 Gy of irradiation. Note: For A, B, and D, the protein bands were quantified using ImageJ software and normalized to β‐actin. Fold changes are shown compared to the negative control without radiation. N/A = not applicable

Next, we studied the role of EGFR‐ERS signaling in radioresistance of OSCC cells by determining the activation of PERK, IRE1α, and ATF6 proteins. EGFR silencing reduced total and phosphorylated PERK, eIF2α, and IRE1α levels as well as splicing of XBP‐1 in the parental and radiation‐resistant FaDu and Detroit562 cell lines upon irradiation. In addition, the 90 kDa ATF6 upon activation translocates to the Golgi apparatus where it gets cleaved by two proteases. And the N terminal 50 kDa ATF6 then works as a transcription factor to induce downstream gene expression. Our results showed EGFR silencing reduced radiation‐induced increases in total and cleaved ATF6 protein in FaDuP and Detroit562P cells, but had no effect in the FaDuR and Detroit562R cells (Figure 3A). These results suggested that EGFR silencing in OSCC cells inhibited the radiation‐induced activation of ERS pathways, namely PERK‐eIF2α and IRE1α‐XBP1, whereas the effects on ATF6 were dependent on the radiosensitivity status of the OSCC cells.

We further assessed the expression of downstream effector proteins of the PERK‐eIF2α and IRE1α‐XBP1 pathways, namely, GRP78, GRP94, PDI, ERO1‐Lα, and CHOP. EGFR silencing inhibited the radiation‐induced expression of the ER molecular chaperones, GRP78 and GRP94, but did not affect the expression of PDI, ERO1‐Lα, and CHOP (Figure 3A). PERK and IRE1α knockdown in OSCC cells resulted in downregulation of GRP94 and GRP78, respectively (Figure 3B). We observed lower PERK and IRE1α mRNA levels in EGFR‐silenced OSCC cells upon irradiation (Figure 3C).

Next, we pre‐stimulated OSCC cells with EGF and observed phosphorylation of PERK, eIF2α, and IRE1α and increased spliced forms of XBP1, GRP78, and GRP94 (Figure 3D). On the other hand, EGF induced ATF6 only in the parental FaDuP and Detroit562P cells (Figure 3D). These results further confirmed that EGF‐EGFR participates in the regulation of the radiation‐induced activation of ERS‐related signaling pathways, that is, PERK‐eIF2α‐GRP94 and IRE1α‐GRP78.

### 3.4 EGFR‐ERS signaling confers radioresistance in OSCC cells by activating DSB repair and autophagy while inhibiting apoptosis

**Figure 4:**
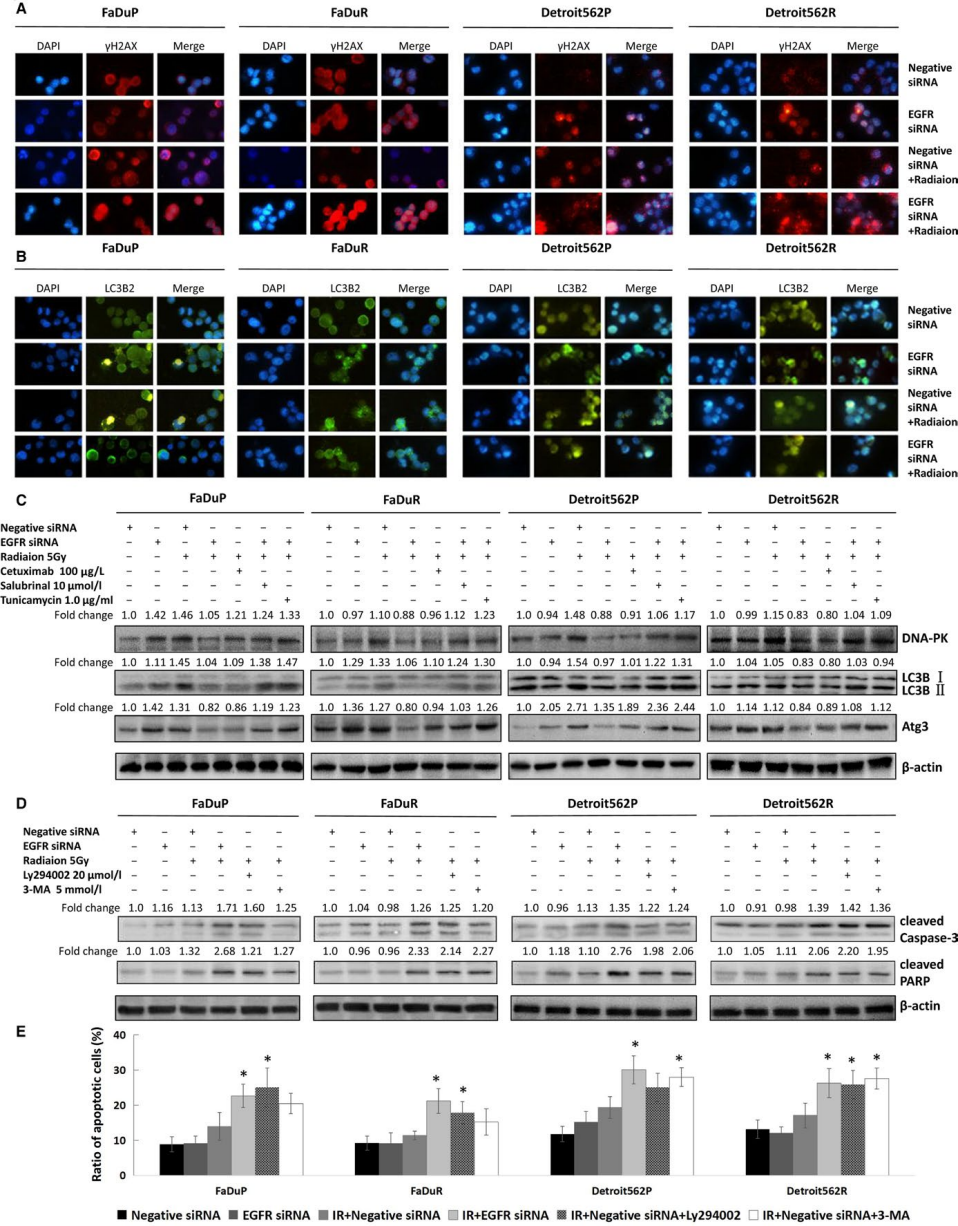
EGFR regulates the radiosensitivity of OSCC cells via ERS and is associated with apoptosis, DSB repair, and autophagy. A, γ‐H2AX foci formation and B, LC3 immunopositive dots in control and EGFR siRNA transfected FaDuR, Detroit562R, FaDuP, and Detroit562P cells followed by irradiation (5 Gy, 2 h). C, DNA‐PKcs, LC3B (LC3B‐II/β‐actin), Atg3, and cleaved caspase 3 levels in control and EGFR siRNA transfected OSCC cells pre‐treated with or without 100 µg/L cetuximab, 10 µmol/L salubrinal, or 1.0 µg/mL tunicamycin for 12 h followed by 5 Gy of irradiation. D, Also shown, cleaved caspase 3 and cleaved PARP in FaDuR, Detroit562R, FaDuP, and Detroit562P cells pre‐treated with or without 20 µmol/L Ly294002 and 5 mmol/L 3‐MA followed by 5 Gy irradiation. E, FACS analysis by AnnexinV/PI double staining of control and EGFR siRNA transfected FaDuR, Detroit562R, FaDuP, and Detroit562P cells, pre‐treated with or without 20 µmol/L Ly294002 and 5 mmol/L 3‐MA followed by 5 Gy irradiation. Note: * denotes *P* < 0.05 compared to the control group

Nagelkerke et al^9^ showed that the inhibition of ERS downregulates radiation‐induced DNA double‐strand break (DSB) repair and increases the radiosensitivity of tumor cells. Thus, we hypothesized that DSB repair mediates EGFR‐ERS regulated radioresistance in oropharyngeal carcinoma cells. Accordingly, immunofluorescence results showed that the combined application of both EGFR silencing and radiation significantly increased γ‐H2AX foci formation which is a marker of the DSB damage (Figure 4A). EGFR knockdown by siRNA as well as treatment with EGFR inhibitor, cetuximab, downregulated radiation‐induced expression of the DSB repair related protein DNA‐PK (Figure 4C). Cotreatment with salubrinal, which induces eIF2α phosphorylation^18^ or the ERS activator tunicamycin after silencing EGFR abrogated EGFR knockdown inhibited DNA‐PK in OSCC cells upon irradiation (Figure 4C).

Autophagy is one of the mechanisms by which tumor cells escape death during starvation and drug treatments, and its inhibition increases the radiosensitivity of esophageal carcinoma cells.^19^ Activation of ERS induces autophagy in nerve cells.^20^ Therefore, we tested the role of EGFR and ERS signaling pathways on radiation‐induced autophagy and observed that EGFR knockdown downregulated the radiation‐induced expression of the autophagy marker protein LC3B and the related pathway protein Atg3, whereas salubrinal and tunicamycin reversed these effects partly (Figure 4B‐C). This suggested that EGFR affects radioresistance via other unknown mechanisms apart from ERS, which need to be studied further.

Our previous study showed apoptosis regulated radioresistance,^21^, ^22^ while the inhibition of DSB repair increased radiation‐induced lymphoma cell apoptosis and radiosensitivity.^23^ In addition, Pang et al^19^ showed that the inhibition of autophagy increased radiation‐induced tumor cell apoptosis. Therefore we analyzed the status of apoptosis and observed that EGFR silencing as well as the DSB repair inhibitor Ly294002 and the autophagy inhibitor 3‐MA increased cleaved caspase 3, cleaved PARP, and AnnexinV^+PI+^ cells (Figure 4D‐E). These results confirmed that EGFR‐ERS signaling inhibited apoptosis, thereby increasing radioresistance of OSCC cells. These effects were reversed by inhibiting autophagy and DSB repair.

### 3.5 EGF‐EGFR increases radiation‐induced cell proliferation by promoting ERK AKT and ERS signaling

**Figure 5:**
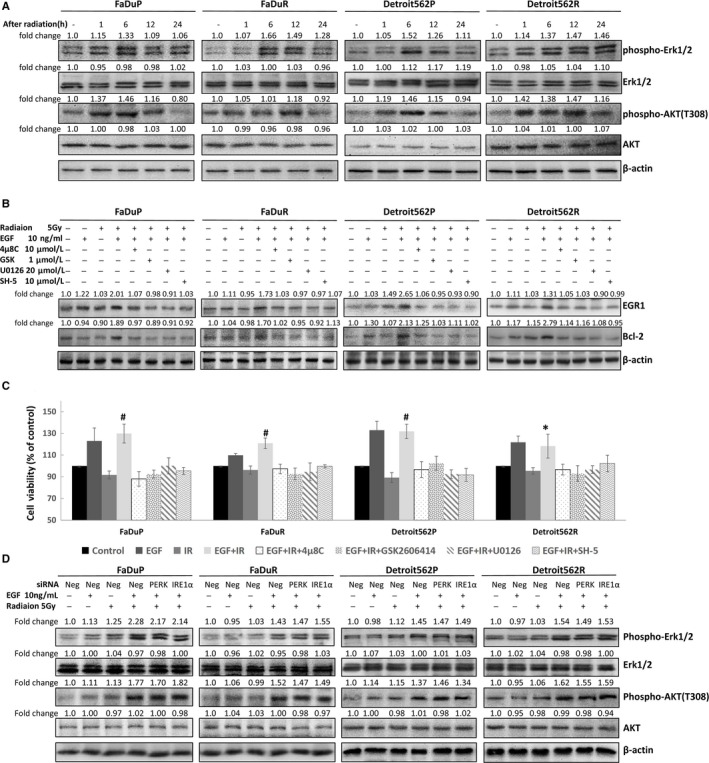
ERS pathway activation regulates EGF‐EGFR‐mediated cell proliferation and radiosensitivity of OSCC cells. A, Total and phosphorylated ERK and AKT at 0‐24 h in FaDuR, Detroit562R, FaDuP, and Detroit562P after 5 Gy of irradiation. B, EGR1 and Bcl‐2 levels in control and EGF (10 ng/mL, 2 h) treatment FaDuR, Detroit562R, FaDuP, and Detroit562P 12 h after 5 Gy of irradiation pre‐treated with or without (IRE1 inhibitor) 4μ8C (10 μmol/L), (PERK inhibitor) GSK2606414 (1 μmol/L), (ERK inhibitor) U0126 (20 μmol/L), and (AKT inhibitor) SH‐5 (10 μmol/L). C, CCK‐8 cell proliferation assay of control and EGF (10 ng/mL, 2 h) treatment FaDuR, Detroit562R, FaDuP, and Detroit562P 48 h after 5 Gy of irradiation pre‐treated with or without (IRE1 inhibitor) 4μ8C (10 μmol/L), (PERK inhibitor) GSK2606414 (1 μmol/L), (ERK inhibitor) U0126 (20 μmol/L), and (AKT inhibitor) SH‐5 (10 μmol/L). Note: * denotes *P* < 0.05, and # denotes *P* < 0.01 compared to the irradiated group. D, Total and phosphorylated ERK and AKT in control and EGF (10 ng/mL) treatment with or without PERK and IRE1α siRNA transfected FaDuR, Detroit562R, FaDuP, and Detroit562P cells 12 h after 5 Gy of irradiation

Next, we analyzed the role of EGF‐EGFR in activating AKT and ERK signaling pathways in OSCC cells upon irradiation. Western blot analysis showed that irradiation increased phosphorylation of AKT and ERK in OSCC cells in a time‐dependent manner (Figure 5A). The phosphorylation peaked at 6 hours and persisted at 24 hours after irradiation in radioresistant FaDuR and Detroit562R cells but decreased to normal levels in the parental FaDuP and Detroit562P cells (Figure 5A). These results suggested that persistent overexpression of the downstream ERK and AKT pathways led to radioresistance in OSCC.

Induction of proliferation and the inhibition of apoptosis are involved in radioresistance.^22^, ^23^ We observed that EGF increased radiation induced the expression of the cell proliferation regulatory protein EGR1 and the anti‐apoptotic protein Bcl‐2 (Figure 5B). This was inhibited by treatment with IRE1 inhibitor 4μ8C, the PERK inhibitor GSK2606414, the ERK inhibitor U0126, and the AKT inhibitor SH‐5 (Figure 5B). CCK‐8 cell proliferation assay showed that combined EGF treatment and irradiation increased the OSCC cell proliferation, but was inhibited by treatment with 4μ8C, GSK2606414, U0126, and SH‐5 (Figure 5C). These results suggested that EGF‐EGFR increased cell proliferation and survival by increasing ERK, AKT and ERS signaling resulting in radioresistance.

To investigate whether the activation of the ERK and AKT pathways was associated with ERS activation, we induced PERK and IRE1α‐silenced OSCC cells with siRNA. Western blot results showed that EGF increased the radiation‐induced phosphorylation of ERK and AKT in PERK and IRE1α‐silenced OSCC cells (Figure 5D). This suggested that AKT and ERK pathway induction by EGF‐EGFR was not mediated by ERS as previously shown by Yu et al.^4^

### 3.6 EGFR and PERK expression in human HPV‐negative OSCC patient tissues

**Figure 6:**
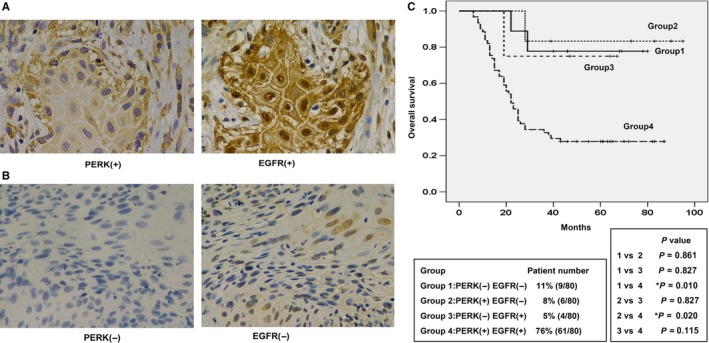
EGFR and PERK expression in human OSCC tissues. Representative images of immunohistochemically stained OSCC patient tissues showing A, high and B, low EGFR and PERK expression with 400× magnification. C, Kaplan‐Meier survival curve analysis showing correlation between the OS and EGFR and PERK expression in 80 OSCC patients

EGFR overexpression is an independent prognostic factor of head and neck cancer and other malignant tumors.^24^, ^25^ Therefore, we analyzed the relevance of EGFR‐ERS signaling in the prognosis of OSCC patients. Immunohistochemical staining of 80 HPV‐negative patient tissue samples showed PERK and IRE1α expression in the cytoplasm, whereas EGFR was expressed in the nucleus and cytoplasm (Figure 6A‐B). Spearman correlation analysis showed that EGFR expression correlated with PERK expression (*r* = 0.378, *P* = 0.001) but did not correlate with IRE1α expression (*r* = 0.209, *P* = 0.097). Kaplan‐Meier analysis of EGFR^−^PERK^−^, EGFR^+PERK−^, EGFR^−^PERK^+^, and EGFR^+PERK+^ patient groups showed that OS was worst in patients co‐expressing both EGFR and PERK (log rank *P* = 0.003, Figure 6C). However, significant differences in survival were not observed among the other groups probably due to small sample sizes.

## 4 DISCUSSION

In recent years, HPV infection has been recognized for its role in the etiology of oropharyngeal carcinoma. Nearly, 70%‐80% OSCC patients in Western countries are infected with HPV,^26^ whereas only 20% are HPV‐positive in China.^27^ Moreover, the etiology, molecular biology characteristics, treatment response, and clinical prognosis vary significantly between HPV‐positive and HPV‐negative OSCC patients.^28^, ^29^ Chung et al^29^ showed that patients with p16‐negative head and neck cancer have worse outcomes than patients with p16‐positive head and neck cancer. Poor prognosis of radiotherapy for HPV‐negative patients was associated with the low radiosensitivity.^30^ Therefore, new strategies are necessary to improve the efficacy of radiotherapy for HPV‐negative OSCC patients. Güster et al showed that EGFR‐targeted therapy did not radiosensitize HPV‐positive OSCC cells.^31^ Therefore, based on the local epidemiological characteristics of the disease, we investigated the molecular pathways that regulate radioresistance in HPV‐negative OSCC cell lines.

EGFR is overexpressed in various tumors including nearly 90% of head and neck tumors.^24^, ^25^ This study showed that 81% HPV‐negative OSCC patients expressed EGFR. Moreover, EGFR overexpression is associated with radioresistance and poor prognosis.^24^, ^25^ We showed that EGFR silencing increased the radiosensitivity of FaDu and Detroit562 cells, especially the radioresistant FaDuR and Detroit562R cell lines, which exhibit stem cell‐like characteristics. One possible explanation of this effect is the higher expression levels of EGFR in radioresistant cells suggesting their overdependence on the EGFR signaling pathway.

Radiation induces DNA damage and ERS to activate UPR, which promotes resistance in some tumors.^10^, ^32^ UPR relies on three transmembrane proteins, PERK, IRE1α, and ATF6 that are localized in the ER PERK is a type I transmembrane ER protein with a ligand‐independent dimerization domain in the N‐terminus that is masked by immunoglobulin protein (BiP)/GRP78 in the absence of ERS and a C‐terminal serine/threonine protein kinase domain without endonuclease activity. ERS activates PERK, which specifically phosphorylates eIF2α at serine 51 to inhibit cellular protein synthesis. We observed that irradiation resulted in EGFR activating PERK, which induced eIF2α phosphorylation and ER chaperone protein GRP94 to regulate the radioresistance of tumor cells, but did not affect CHOP, which is downstream of PERK. These results confirmed previous findings that moderate activation of PERK did not induce CHOP.^7^

Activation of the ER splicing factor IRE1α, which splices the transcription factor XBP1, induces chaperones that are necessary to increase protein folding and modulate mRNA degradation and translation.^33^ Yu et al^4^ showed that EGFR activation was necessary for the activation of IRE1α. We confirmed that EGFR silencing inhibited radiation‐induced IRE1α‐XBP1 expression, which further inhibited GRP78.

ATF6 activates the gene promoter region of stress elements in the ER Subsequently, these genes activate the transcription of molecular chaperones, foldases, and CHOP. Yu et al^4^ showed that silencing ATF6α inhibits EGF‐induced breast cancer cell proliferation. We showed that EGFR silencing inhibited radiation‐induced ATF6 expression in radiosensitive OSCC cells; however, this effect was cell specific. In addition, we confirmed that PERK‐eIF2α and IRE1α‐XBP1 activation blocked apoptosis and promoted proliferation without affecting activation of the ERK and AKT signaling pathways.

In addition to the three conventional ERS pathways that are induced by EGFR upon X‐ray irradiation, EGF also activates the anticipatory UPR by releasing ER calcium stores at an early stage after EGF‐EGFR signaling (<20 minutes). Subsequently, GRP78 expression is increased by ERS activation‐induced UPR.^4^ In the present study, we demonstrated that EGFR silencing suppressed activation of PERK‐eIF2α‐GRP94 and IRE1α‐XBP1‐GRP78 pathways for 12 hours after irradiation. Based on the time frame, we postulate EGFR activated the conventional ERS‐UPR pathways upon irradiation and did not involve the anticipatory UPR.

Autophagy is a current focus of radiotherapy resistance studies. Both radiotherapy and chemotherapy generate enormous intrinsic cellular stress as a result of damaged proteins and organelles, which induces autophagy to repair DNA and other cellular damage to restore cellular homeostasis.^34^ Moreover, autophagy inhibition increased the anti‐tumor effects of cetuximab.^35^ Also, EGFR‐targeted therapy increased the radiosensitivity of non‐small cell lung cancer by inhibiting autophagy.^36^ Interestingly, ERS also induces autophagy.^20^, ^37^ The regulation of radiosensitivity by autophagy is pleiotropic. Recently, Li et al^38^ showed that silencing CHOP inhibited radiation‐induced autophagy, thereby increasing the radiosensitivity of MDA‐MB‐231 breast cancer cells. The inhibition of apoptosis also produced radioresistance in MCF‐7 cells.^38^ In contrast, JNK silencing inhibited radiation‐induced autophagy and increased the radiosensitivity of MCF‐7 cells.^38^ Our study showed that irradiation of EGFR‐silenced OSCC cells decreased the expression of the cell autophagy‐related proteins, LC3B and Atg3. Moreover, treatment with the eIF2α phosphorylation activator, salubrinal, and the ERS activator, tunicamycin, abrogated this effect. In addition, treatment with the autophagy inhibitor 3‐MA enhanced radiation‐induced apoptosis in EGFR‐silenced OSCC cells. Taken together, our study showed that enhanced radiosensitivity of EGFR‐silenced OSCC cells might be facilitated by ERS‐induced autophagy.

Exposure to ionizing radiation activates many stress signaling pathways that induce DNA repair, maintain cell proliferation, and inhibit apoptosis. These mechanisms lead to radioresistance. The inability to repair DNA is closely associated with increased radiosensitivity of tumor cells. Under normal conditions, radiation‐induced DSBs are repaired for cell survival. If DNA damage is left unrepaired, cells activate pathways of programmed cell death. Inhibition of the PERK/ATF4/LAMP3 pathway increased radiosensitivity of breast cancer by interfering with DNA damage repair.^9^ Moreover, EGFR‐targeted therapy increased the radiosensitivity of non‐small cell lung cancer by inhibiting DSB repair.^36^ ATM is a critical protein that senses radiation‐induced DNA damage, gets phosphorylated, and activates downstream target proteins that inhibit cell cycle progression until completion of DNA repair or initiation of apoptosis.^39^ Our previous studies showed that ATM phosphorylation induced G2/M phase arrest in lymphoma cells and increased their radiosensitivity.^23^ Proficient DSB repair is facilitated by activating the DSB repair enzyme, DNA‐dependent protein kinase, and its catalytic subunit (DNA‐PKcs).^40^ Mukherjee et al^41^ showed that EGFRvIII‐induced DNA‐PKcs regulate radioresistance in glioblastoma. EGFR silencing inhibited radiation‐induced DNA‐PK expression, and these effects were inhibited by salubrinal and tunicamycin. In addition, Ly294002 treatment increased radiation‐induced OSCC cell apoptosis. Therefore, we demonstrated that increased radiosensitivity of EGFR‐silenced OSCC cells was mediated by the inhibition of radiation‐induced DNA double‐strand break repair by ERS that increased apoptosis.

Previous studies showed that overexpression of EGFR^24^, ^25^ as well as ERS chaperone protein GRP78^11^ was associated with poor prognosis in head and neck squamous cell carcinoma patients. However, the correlation between EGFR and ERS signaling pathways in head and neck squamous cell carcinoma has not been well studied. We demonstrated by immunohistochemical analysis that EGFR and PERK overexpression was associated with a poor response to radiotherapy in OSCC (Figure 5C).

**Figure 7:**
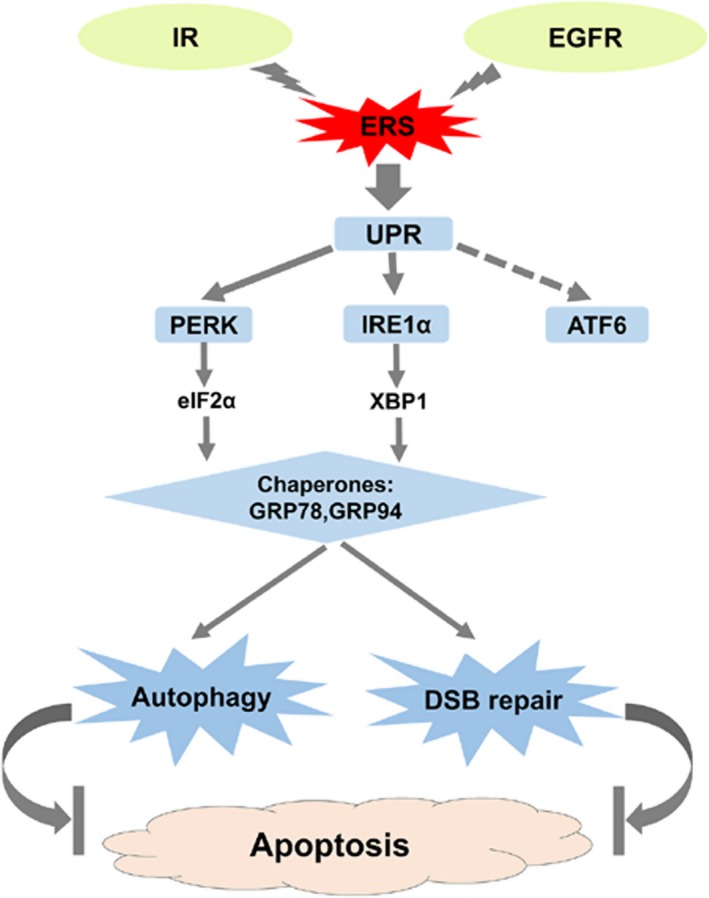
Schematic representation of radiation‐induced ERS signaling pathways mediated by EGFR related to radioresistance

In conclusion, our study demonstrated that irradiation induced time‐dependent increase in EGFR expression, which promotes radioresistance of OSCC cells by activation of the ERS‐related pathways, PERK‐eIF2α‐GRP94 and IRE1α‐XBP1‐GRP78. EGFR knockdown inhibited radiation‐induced ERS, autophagy, and DSB repair pathways, thereby promoting radiation‐induced cell apoptosis (Figure 7). OSCC patients with co‐expression of EGFR and PERK proteins showed poor prognosis, thereby highlighting their therapeutic potential for OSCC patients.
